# The genetic analysis of tolerance to infections: a review

**DOI:** 10.3389/fgene.2012.00262

**Published:** 2012-12-14

**Authors:** Antti Kause, Jørgen Ødegård

**Affiliations:** ^1^Biotechnology and Food Research, Biometrical Genetics, MTT Agrifood Research FinlandJokioinen, Finland; ^2^NofimaÅs, Norway

**Keywords:** cure model, genotype-by-environment interaction, mixture model, quantitative genetics, random regression, resistance, statistical methods, tolerance

## Abstract

Tolerance to infections is defined as the ability of a host to limit the impact of a given pathogen burden on host performance. Uncoupling resistance and tolerance is a challenge, and there is a need to be able to separate them using specific trait recording or statistical methods. We present three statistical methods that can be used to investigate genetics of tolerance-related traits. Firstly, using random regressions, tolerance can be analyzed as a reaction norm slope in which host performance (*y*-axis) is regressed against an increasing pathogen burden (*x*-axis). Genetic variance in tolerance slopes is the genetic variance for tolerance. Variation in tolerance can induce genotype re-ranking and changes in genetic and phenotypic variation in host performance along the pathogen burden trajectory, contributing to environment-dependent genetic responses to selection. Such genotype-by-environment interactions can be quantified by combining random regressions and covariance functions. To apply random regressions, pathogen burden of individuals needs to be recorded. Secondly, when pathogen burden is not recorded, the cure model for time-until-death data allows separating two traits, susceptibility and endurance. Susceptibility is whether or not an individual was susceptible to an infection, whereas endurance denotes how long time it took until the infection killed a susceptible animal (influenced by tolerance). Thirdly, the normal mixture model can be used to classify continuously distributed host performance, such as growth rate, into different sub-classes (e.g., non-infected and infected), which allows estimation of host performance reduction specific to infected individuals. Moreover, genetics of host performance can be analyzed separately in healthy and affected animals, even in the absence of pathogen burden and survival data. These methods provide novel tools to increase our understanding on the impact of parasites, pathogens, and production diseases on host traits.

## Introduction

**Figure 1:**
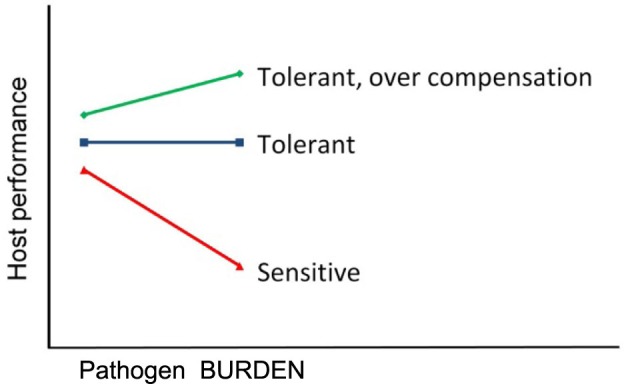
**Tolerance to infections**. Tolerance is the reaction norm slope of host performance regressed against individual's pathogen burden. The lines represent performance of three genotypes with a different degree of tolerance.

Tolerance and resistance are two different defense mechanisms to defend against pathogens and parasites. Resistance is the ability of a host to prevent pathogen entry and to control pathogen life cycle in a way to reduce pathogen burden within a host individual. Tolerance to infections, in turn, is defined as the ability of the host to limit the impact of a given pathogen burden on host health, performance, and ultimately on host fitness (Clunies-Ross, 1932; Painter, 1958; Albers et al., 1987; Simms and Triplett, 1994; Simms, 2000) (Figure 1).

Being able to uncouple resistance and tolerance is essential for several reasons. Firstly, they have different impact on the arms-race co-evolution between the host and the pathogen (Mauricio et al., 1997; Rausher, 2001; Bishop and MacKenzie, 2003; Best et al., 2008). Moreover, both in animals and plants, tolerance and resistance are weakly genetically correlated, and thus they are genetically different traits (Leimu and Koricheva, 2006; Ødegård et al., 2011b; Kause et al., 2012). Finally, animal and plant breeders should exploit both increased resistance and tolerance to ensure global food security.

In addition to pathogens, tolerance can be assessed against abiotic factors such as temperature, heavy metals, or against production diseases causing damage to body tissues (Ravagnolo and Misztal, 2000a,b; Schat et al., 2002; Bloemhof et al., 2012; Kause et al., 2012). Naturally, production diseases, such as ascites, are not standard disease traits caused by a pathogen or parasite infection. Thus, there is no co-evolution between a host and a production disease, and the production disease does not evolve in response to the evolution of the host. Nevertheless, improved resistance and tolerance can be both used to reduce the harmful effects of production diseases on farmed animals, motivating their tolerance analysis (Kause et al., 2012). From hereon in this paper, pathogen burden is used as a general term to refer to a pathogen load of an individual, for instance, number or biomass of ecto- and endoparasites, number of pathogens in a blood sample, or severity of a production disease. In plants, pathogen burden may refer to the biomass or number of herbivores, or percentage of leaf area lost to herbivores.

The objective of this paper is to present recent statistical advances in the genetic analysis of tolerance-related traits. Firstly, random regression models have been applied to tolerance analysis. They allow a sophisticated genetic analysis of traits defined as functions as well as the quantification of genotype-by-environment interactions (G × E) induced by infections (Kause, 2011; Kause et al., 2012). Secondly, Ødegård et al. (2011b,c) introduced a cure model to separate “susceptibility” and “endurance” from challenge test data with time-until-death observations (without having any knowledge about infection status of the animals). The first trait is comparable to resistance, while endurance may be influenced by tolerance. “Susceptibility” can be defined as whether or not the animal is liable to die as a result of an infection (i.e., long-term survival, which is likely associated with resistance), while “endurance” is defined as how long time it takes before a potential infection kills the animal (which is likely associated with tolerance). Both endurance and susceptibility may show genetic variation, and may be viewed as different genetic factors affecting survival under an infection. Finally, normal mixture models can be extended to involve responses in host performance traits (e.g., growth curves) specific to healthy and affected individuals (Wang and Bodner, 2007; Madsen et al., 2008).

## Random regression models

Tolerance is by definition the change in host performance as a function of pathogen burden (Simms, 2000), and hence, it is natural to apply random regression models to estimate genetic parameters and breeding values for tolerance (Kause, 2011). Using random regressions, tolerance can be analyzed as a reaction norm in which host performance (on *y*-axis) is regressed against pathogen burden of individuals (on *x*-axis) (Box 1). It is important to note that pathogen burden is measured separately from each individual, and it is not a general environmental characteristic. The slope of such a regression is consistent with the definition of tolerance (Figure 1), and hence genetic variance in regression slopes is the genetic variance for tolerance (Kause, 2011).

Box 1 **A random regression model.**

An animal model random regression model is of the form:

*y*_*i*_ = *b*_0_ + *b*_1_*PBurden* + *b*_0*i*_ + *b*_1*i*_*PBurden*_*i*_ + ϵ_*i*_, where *y*_*i*_ is host performance of an individual *i* at its pathogen burden *PBurden*, *b*_0_ is the fixed population mean intercept, *b*_1_* PBurden* is the fixed population mean tolerance slope, *b*_0*i*_ is the random genetic effect of intercept for an individual *i*, *b*_1*i*_PBurden_*i*_ is the random genetic effect of tolerance slope for an individual *i*, and ϵ_*i*_ is the random error term. Both *b*_0*i*_ and *b*_1*i*_ are modeled with a pedigree, allowing the estimation of their genetic variance.

**Covariance functions**. Genetic variance of host performance as a function of pathogen burden can be calculated: as *x*'_*PBurden*_**G***x*_*PBurden*_, where $G=\left[\begin{array}{cc} \sigma_{b0}^{2} & \sigma_{b0b1}\\ \sigma_{b0b1} & \sigma_{b1}^{2} \end{array}\right]$, σ^2^_b0_ and σ^2^_b1_ are genetic variances for intercept and slope, respectively, and σ_b0b1_ is covariance between the two terms (Kolmodin and Bijma, 2004). The term *x*_*PBurden*_ is a vector [1 *PBurden*]′ in which *PBurden* refers to a pathogen burden value on the *x*-axis. A genetic correlation between the performance of non-infected (*PBurden* = 0) and infected individuals at a certain *PBurden* value can be calculated as: $r_{G}=\frac{x_{0}^{\prime }Gx_{PBurden}}{\sqrt{x_{0}^{\prime }Gx_{0}\;\times \;x_{PBurden}^{\prime }Gx_{PBurden}}}$, where **G** is the genetic (co)variance matrix of slope and intercept, *x*_0_ is a vector of [1 0]′, and *x*_*PBurden*_ is as described earlier (Calus et al., 2004).

The intercept of the tolerance regression is interpreted as the host performance in a pathogen-free environment, and the genetic correlation between the slope and the intercept quantifies the degree to which host performance under no infection is genetically traded off with tolerance. Moreover, genetic correlations of the slope and intercept with third-party traits can be estimated by extending the random regression model to multitrait animal or sire model (Kause et al., 2012).

In animals, pathogen burden is typically a continuously distributed trait, especially when a population is under a natural pathogen infection (Stear et al., 1995; Kuukka-Anttila et al., 2010). Even in a challenge test in which all individuals are exposed to the same initial pathogen load, variation among individuals in resistance creates continuous variation in pathogen burden. Random regression models allow genetic analysis of tolerance along a continuous pathogen burden trajectory. In animal breeding, random regression models have been commonly applied to the reaction norm analysis of G × E (Henderson, 1982; Meyer and Hill, 1997; Calus et al., 2004; Schaeffer, 2004; Lillehammer et al., 2009).

### Tolerance-induced variation in host performance

Genetic variation in tolerance may induce G × E in host performance, leading to changes in genetic variation of host performance along an increasing pathogen burden trajectory. For instance, in Figure 1, genetic variance in host performance is elevated along increased pathogen burden due to diverging tolerance reaction norms. In poultry, pigs, and aquaculture species, breeding nucleuses may be held infection-free due to biosecurity reasons, whereas commercial production and/or collection of sib and progeny information for breeding value estimation occurs at field farms with diverse diseases present. Such a design may induce G × E due to variation in the level of tolerance, which should be accounted for in breeding value evaluations.

In an infection-free environment, individual variation in host performance, e.g., in growth rate, is due to variation in genetic potential for growth and unexplained environmental variation. Under infection, in turn, individual variation in both resistance and tolerance induce additional variation into host performance. Some individuals are fully resistant or are not exposed to an infection, and thus their growth is not influenced by the infection. Some individuals are infected, and the degree to which their growth rate is reduced depends on their pathogen burden and the level of tolerance. Growth of fully tolerant individuals is not affected, whereas growth of very sensitive ones is greatly reduced.

**Figure 2:**
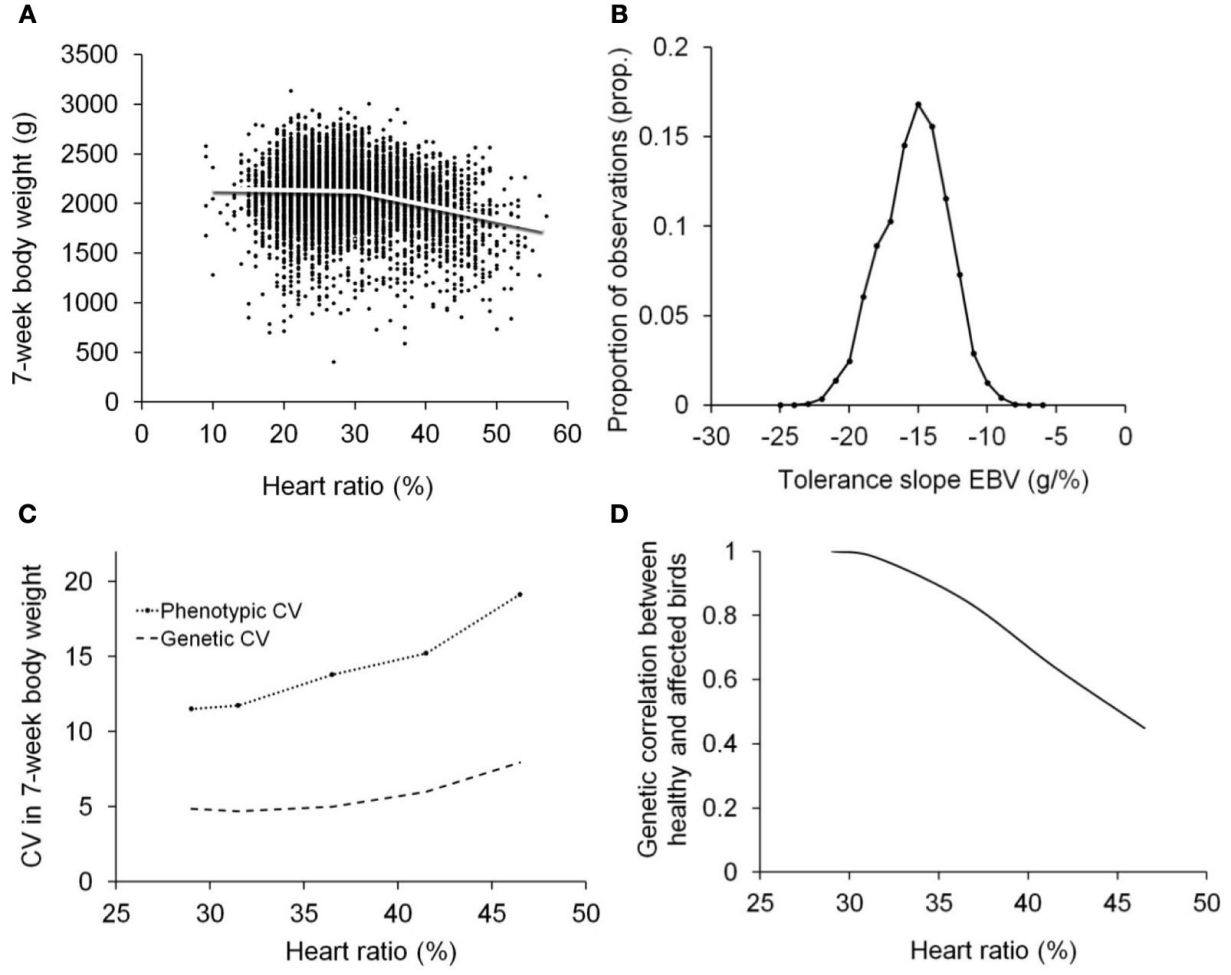
**Tolerance analysis using random regressions and covariance functions illustrated using data on 7-week body weight and heart ratio of broilers [reproduced from Kause et al. (2012); http://creativecommons.org/licenses/by/3.0/]**. Heart ratio, the ratio of right ventricular weight to total heart weight, is an indicator of ascites resistance, the birds with higher than 27–30% heart ratio typically being ascitic (Wideman et al., 1998). Tolerance is the change in body weight as a function of increasing ascites severity, measured as the hearth ratio. **(A)** Population average tolerance curve with body weight on *y*-axis and ascites severity on *x*-axis. **(B)** Frequency distribution of estimated breeding values for tolerance slopes of individuals, showing sensitive (steep negative slope) and more tolerant (weak negative slope) genotypes. **(C)** Increased coefficients of phenotypic and genetic variation in body weight as a function of ascites severity, showing ascites molds trait variation. **(D)** Genetic correlation between healthy birds and birds with different degree of ascites severity, showing ascites creates genotype re-ranking (Kause et al., 2012).

Despite the large number of studies dealing with the changes induced by biotic (e.g., diet) and abiotic factors in general (Hoffmann and Merilä, 1999; Kause and Morin, 2001; Charmantier and Garant, 2005), there has been only a limited focus on infection-induced changes in genetic parameters and the consequent environment-specific genetic responses to selection (van der Waaij et al., 2000). Infections are indeed known to induce changes in heritability of host performance traits (Charmantier et al., 2004; Pakdel et al., 2005; Zerehdaran et al., 2006; Kause et al., 2007, 2012; Vehviläinen et al., 2008; Lewis et al., 2009). Yet, currently we do not know how much of the phenotypic variation in host performance is in fact created by infections and the associated tolerance. A study by Kause et al. (2012) showed that coefficient of phenotypic variation in broiler body weight was elevated from 11.5% when birds were healthy, to 19.1% when birds were severely affected by ascites. Similarly, coefficient of genetic variation was increased from 4.9% to 7.9%, implying the changes in variance can be extensive (Figure 2). It is hypothesized that in populations exposed to infections, a large proportion of phenotypic variance in host traits is induced by infections and the associated individual variation in resistance and tolerance.

Random regression models combined with covariance functions (Kirkpatrick et al., 1990; Meyer and Hill, 1997) provide means to quantify the changes in phenotypic and genetic variances in host traits along a continuous pathogen burden trajectory (Kause, 2011; Kause et al., 2012). Given the genetic (co)variance estimates of tolerance slope and intercept estimated using random regressions, the changes in genetic variance in host performance can be calculated using formulas (Box 1; Figure 2). The same logic can be applied to the maternal and environmental components of (co)variance.

### Tolerance-induced genotype re-ranking in host performance

Crossing tolerance reaction norms create genotype re-ranking in host performance traits across pathogen burden trajectory. This is similar to any genotype re-ranking across environmental gradients (Via and Lande, 1985), with the difference that now the environment is pathogen burden of individuals (Kause et al., 2012). The two forms of G × E, scaling effect and genotype re-ranking, facilitate environment-dependent genetic responses, yet the re-ranking is more severe issue for selective breeding because genotypes in one environment are not necessarily the best ones in the other environments. Re-ranking across environments can be quantified by a genetic correlation between measurements in two environments for a given trait (Falconer, 1952).

The degree of re-ranking between any two pathogen burden levels can be calculated by combining random regression results with covariance functions (Box 1). For instance, ascites induced moderate genotype re-ranking in broiler body weight, the genetic correlation of healthy birds with weakly affected birds being unity but with severely affected birds 0.45 (Kause et al., 2012; Figure 2). In field data sets with multiple environments, infection pressure is typically not the only environmental factor varying across environments, yet the effect of pathogen burden on G × E could be revealed using a combined reaction norm and multi-trait model (Windig et al., 2011), in which pathogen burden is modeled as a continuous reaction norm and the discrete environments capturing other environmental factors are modeled as separate discrete traits. Performing extensive infection-challenge tests is impractical in many farm animal species, but the combined reaction norm and multi-trait model may be an effective additional method for revealing the degree of G × E induced by infections.

Infections do not induce only genotype re-ranking and a change in variance but also changes in the correlation structure of resistance, growth, and reproduction traits (de Greef et al., 2001; Kause et al., 2005, 2012; Zerehdaran et al., 2006; Kuukka-Anttila et al., 2010). The modification of genetic architecture of host traits by pathogens, parasites, and production diseases, mediated by tolerance genetics, may play a more fundamental role in animal breeding and microevolution than has been previously thought.

### Data requirements for random regression

Obtaining a solid *x*-axis is a major challenge for the tolerance analysis in animals because the *x*-axis should consists of individual-level quantitative data on pathogen burden (e.g., number of parasites, pathogen biomass). Qualitative data on burden (infected vs. non-infected individuals) creates biased estimates of genetic variance for tolerance (Kause, 2011). Moreover, if the *x*-axis consists of the average burdens of each environment, rather than individual-level burden measurements, then high host performance of a genotype at a given pathogen burden can be a result of high resistance and/or high tolerance, impeding a proper tolerance analysis. The analyzed host performance trait, in turn, can be feed intake, growth, reproduction, survival or a physiological trait, which together can be used to reveal mechanisms contributing to variation among genotypes in tolerance.

A split-family design with both an infection-free control and an experimental challenge test is the most effective design for tolerance analysis. In this way, infected animals are a random sample of their family and thus there will be a real causal relationship between host performance and pathogen burden (Tiffin and Inouye, 2000; Kause, 2011). This requires, however, that all the challenged individuals get the same pathogen burden level. This rarely is the case because individuals have innate individual variation in resistance, creating variation in pathogen burden even in a challenge test. Variation in resistance can be potentially related to the host performance traits used on the *y*-axis in the tolerance regression, biasing the estimate of genetic variation for tolerance (Tiffin and Inouye, 2000; Kause, 2011). As an alternative to the control-and-challenge test design, all individuals can be first recorded under infection-free conditions (e.g., for mature body weight), and then re-recorded after experimental exposition to equal pathogen burden level. However, such an analysis is unjustified in cases in which host performance shows natural temporal variation (e.g., variation in growth curves), which is thus confounded with tolerance (Albers et al., 1987; Bisset and Morris, 1996; Woolaston and Windon, 2001). Trypanotolerance of African cattle has been analyzed as a change in body weight in response to an experimental infection by *Trypanosoma congolense*, but although the number of parasites in the blood of individuals was recorded, it was not used to standardize the host performance changes of individuals (Hanotte et al., 2003; van der Waaij et al., 2003).

Under naturally occurring infection, it is possible that either high (or low) performing individuals are infected, leading to biased estimates of genetic variation for tolerance (Tiffin and Inouye, 2000; Kause, 2011). This is a major weakness of field data sets, because it is well established that individuals with initially different growth or life-history trait levels may be differently exposed to infections, parasites and production diseases (Arendt, 1997; Rauw et al., 1998), confounding the cause-and-effect relation between pathogen burden and reduction in host performance.

Random regression models require large sample sizes, e.g., within sire families. Decrease in family size leads to upward-biased genetic variance estimates for tolerance slope (Kause, 2011). This can be illustrated in a sire model set up. When a small number of individuals are sampled for each sire family, the sample is no longer representative of the true distribution and single observations have strong impact on the slope estimate. For some families the slope is underestimated, for others overestimated, and thus genetic variance estimate for slope is artificially increased. With heritability of 0.3 for tolerance slope, more than 50 sibs per family are required to obtain unbiased estimates of slope variance using a sire model analysis (Kause, 2011). Moreover, genetic correlation between tolerance slope and intercept is easily biased downward when family size is low. An upward (downward) bias in the slope of a family pushes the intercept downward (upward), creating an artificial negative genetic trade-off when it does not exist in reality. This can be avoided by using high family sizes and high number of non-infected individuals that force the intercept of a genotype to be placed close to the real value (Mauricio et al., 1997; Kause, 2011).

When each host individual has only a single performance record, it is possible to estimate genetic variance and breeding values for tolerance slope, but not its residual variance. Heritabilities for tolerance slope can be estimated when each individual has several performance observations, e.g., the initial performance under conditions of no infection and thereafter the performance after an infection. By using regression slopes of individuals as raw observations in the genetic analysis, both environmental and genetic components of slope variance and heritability can be estimated (Schaeffer, 2004).

Random regression can be applied to non-linear reaction norms (Kirkpatrick et al., 1990; Meyer and Hill, 1997; Schaeffer, 2004) and plateau-linear regression models (Ravagnolo and Misztal, 2000a,b; Kause et al., 2012), and thus the impact of pathogens on host performance does not need to be analyzed as a linear relationship.

## A cure model for time-until-death data

The random regression approach requires individual-level data on pathogen burden which may be challenging to record. The cure model for time-until-death data provides a possibility to analyze genetics of resistance (or susceptibility) and endurance without a need for pathogen burden recording.

**Figure 3:**
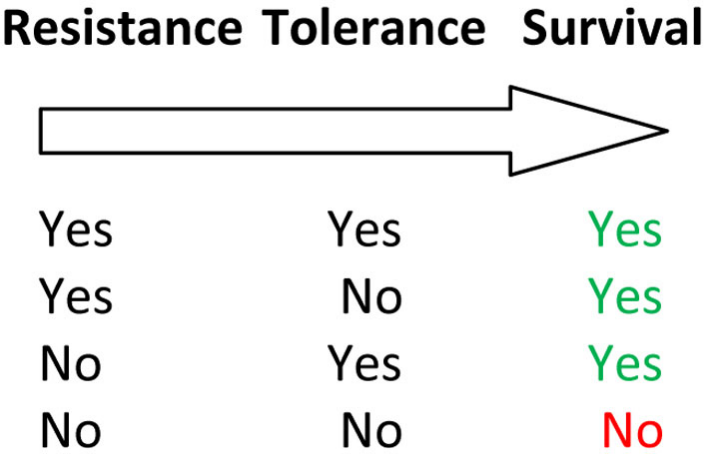
**Contribution of resistance and tolerance to mortality due to a specific pathogen**. Only individuals without resistance and tolerance will eventually die given a sufficiently long follow-up period. When having a limited follow-up period, individuals with high tolerance may still be alive at the end of an experiment.

Many studies, especially on aquaculture species, have analyzed survival or time-until-death in a challenge test in which individuals are experimentally exposed to a specific pathogen (Ødegård et al., 2011a). Moreover, survival analysis has been applied to time-until-death data when mortality factors remain unknown (e.g., Ducrocq and Casella, 1996; Serenius and Stalder, 2004; Vehviläinen et al., 2010). A typical assumption in such analyses is that individuals with high probability of survival are resistant. However, an individual can survive if it has either high resistance, or low resistance but high tolerance (Figure 3), or was never exposed to a pathogen. The cure survival models are used for modeling of time-until-death data which include a fraction of non-susceptible animals, i.e., animals that are not liable to die as a result of the infection (Farewell, 1982). Ødegård et al. (2011c) developed a cure model aiming to distinguish two traits, “susceptibility” and “endurance,” from time-until-death data. These two concepts may be comparable with resistance and tolerance.

In a survival analysis the infection status of each animal is typically unknown. Under pathogen attack, some animals may be fully capable of avoiding death (non-susceptible), either by resisting the infection, or by a successful recovery after the initial infection due to high tolerance (Figure 3). Furthermore, the degree of tolerance may also vary among the susceptible individuals, potentially causing variation in their expected time-until-death. As mortality is usually recorded over a limited follow-up period, a fraction of susceptible animals are also likely to be alive at the time of recording. For susceptible animals, the ability to survive depends on the expected time-until-death of the animal, which may show genetic variation. Hence, analogy of the terms “endurance” and “susceptibility” with tolerance and resistance are not necessarily clear-cut in a survival analysis, due to the fact that one only observes the extreme outcomes of an infection (whether or not an animal dies). Although “endurance” and “susceptibility” are impossible to separate on individual survivors, these two factors may still be distinguished on a family level using longitudinal survival analysis (i.e., short-term mortality rates vs. long-term survival).

A classical survival analysis of time-until-death assumes that all individuals are at risk and that all will eventually die given a sufficiently long follow-up period. When studying lifespan in general this is necessarily true, but may not hold when testing for mortality due to a specific pathogen. For non-susceptible animals time-until-death will necessarily be censored, irrespective of the follow-up time, and survival time may thus be a poor indicator for specific pathogen resistance. The endurance reflects the expected mortality per time-unit among susceptible individuals, but will have no effect on survival of the non-susceptible individuals (Farewell, 1982).

The survivors are likely a mixture of non-susceptible long-term survivors and a fraction of susceptible (but highly endure) animals being still alive, and the true condition of each animal is unknown (unless the animal dies). In the cure model, probabilities of the alternative settings (non-susceptible or susceptible but still alive) can be estimated while simultaneously taking into account variation in endurance among the surviving animals (Ødegård et al., 2011c; Box 2).

Box 2 **A cure model.**

In a mixed population of susceptible (*z* = 1) and non-susceptible (*z* = 0) animals the probability for an individual being still alive (censored) (*c* = 0) at time t is:

$$\begin{array}{l} Pr(c=0|t)=Pr(c=0|t,\;z=1)Pr(z=1)+Pr(c=0|t,\;z=0)Pr(z=0)\\ \;=Pr(c=0|t,\;z=1)Pr(z=1)+\left(1-Pr(z=1)\right), \end{array}$$

where *Pr*(*z* = 1) is the prior probability of being susceptible and *Pr*(*c* = 0|*t*, *z* = 0) = 1. The probability of being still alive for susceptible animals, *Pr*(*c* = 0|*t*, *z* = 1), is a function of the endurance of the animal. Furthermore, if survival time is split into a series of binary survival scores (e.g., s_1_ to s_*t*_, where 0 indicates survival), this probability is:

$$Pr(c=0|t,\;z=1)=\prod_{j=1}^{t}Pr(s_{j}=0|z=1),$$

where *Pr*(*s*_*j*_ = 0|*z* = 1) is the probability of surviving a period *j*, given that the animal is susceptible. Highly endure animals will have higher probabilities of surviving each sub-period and thus also higher probability of surviving until end of follow-up period. Putative non-susceptible animals will always survive.

For animals that die during the follow up period, susceptibility status is known (*z* = 1), while for surviving animals the true susceptibility status is not observable. Still, for these individuals the probability of being susceptible can be calculated as:

$$Pr(z=1|c=0,\;t)=\frac{Pr(c=0|t,\;z=1)Pr(z=1)}{Pr(c=0|t)}$$

The proposed cure model allows for individual variation in both prior probability of being susceptible as well as in the endurance of susceptible animals (Ødegård et al., 2011b,c). A detailed description of the cure model is given in Ødegård et al. 2011c.

The cure model has been applied to time-until-death data in farmed shrimp challenge-tested with the Taura syndrome virus (Ødegård et al., 2011b). It was estimated that although 72% of the shrimp survived, only 62% could be considered non-susceptible. The underlying heritability (±SE) for susceptibility was high (0.41 ± 0.07), while the heritability of endurance was low, albeit significant (0.07 ± 0.03). The most striking result was that endurance and susceptibility were seemingly distinct genetic traits (*r*_G_ = 0.22 ± 0.25). The low genetic variation for endurance and the genetic independency of endurance and susceptibility are in line with the results on other animal species (Kause et al., 2012). These results have substantial impact on how disease challenge-testing should be performed. If the aim is to improve long-term survival under an infection pressure, selective breeding should focus on susceptibility. This implies that the follow-up period should continue until the vast majority of susceptible animals have died, ensuring that the observed end-survival largely resembles the fraction of non-susceptible animals in the population.

## Normal mixture models

Normal mixture models can be used to analyze genetics of host performance, e.g., growth rate, within a population consisting of individuals affected and unaffected by a pathogen, even in the absence of pathogen burden and time-until-death data.

**Figure 4:**
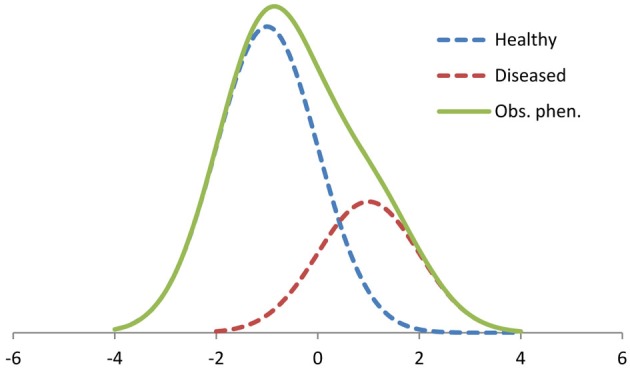
**An example of a two-component mixture distribution**. The dotted lines are the unobserved distributions of non-infected “healthy” individuals (70% of the observation) with ~*N*(−1.0, 1.0) and infected “diseased” individuals (30%) with ~*N*(1.0, 1.0). The solid line represents the resulting distribution of the observed phenotypes. Trait values are given on *x*-axis and the frequencies of observations on *y*-axis.

Finite normal mixture models have earlier been proposed for analysis of infection-affected, continuously distributed phenotypes, assuming that the true infection statuses of individuals are unknown (Detilleux and Leroy, 2000; Ødegård et al., 2003, 2005; Gianola et al., 2004). The mixture model attempts to identify hidden categories (e.g., non-infected and infected) among the observations, assuming that the continuous scale observations originate from two normal distributions differing in mean and (potentially) variance (Box 3; Figure 4). For instance, the broiler ascites example given in Figure 2 can be analyzed using a mixture model analysis assuming that the heart ratio has two underlying distributions, one for non-infected and one for ascitic birds (Zerehdaran et al., 2006). Another example of a mixture trait is somatic cell scores in milk of dairy cattle (Madsen et al., 2008). Somatic cell score is at low level in non-infected cows, but increase to high levels in cases of (unobserved) subclinical mastitis. Hence, the observed somatic cell scores may be viewed as a mixture of two normal distributions (non-infected and mastitic). In Atlantic salmon *Salmo salar* L., diseases such as infectious pancreas necrosis and pancreas disease can kill a fraction of the animals, but may also reduce subsequent growth of affected survivors. Hence, after an outbreak, observed growth of survivors may be viewed as a mixture trait depending on the individuals' previous health status.

Box 3 **A normal mixture model.**

In a mixed population of infected (*z* = 1) and healthy (*z* = 0) animals, the density of an observation *y* can be written as:

P(y)=P(y|z=0)Pr(z=0)+P(y|z=1)Pr(z=1).

The probability of an animal being infected is thus:

$$Pr(z=1|y)=\frac{P(y|z=1)Pr(z=1)}{P(y|z=0)Pr(z=0)+P(y|z=1)Pr(z=1)}\cdot$$

A detailed description of the normal mixture model is given in Ødegård et al. (2003, 2005).

Classical selection aims at changing a trait in the desired direction. However, for mixture traits the variation is partly explained by mixing of the two (or more) sub-distributions with different means, and partly by variation within each sub-distribution (Figure 4). Hence, if the aim is to reduce the incidence of the infection rather than altering the observed continuous host trait itself, simple directional selection for the latter (e.g., for somatic cell score) may not be optimal. The mixture model opens new possibilities for selection, and can be used to directly select for reduced infection risk. Additionally, the trait recorded on infected and non-infected animals may be viewed as two distinct sub-traits whose genetic variances and their genetic correlation can be estimated. This resembles the G × E analysis performed with random regression models (Figure 2) with the difference that the mixture model does not take into account that infected individuals may have different pathogen burdens.

Normal mixture models typically assume that an individual is either infected or not, and that infection has a certain effect on the phenotype (Figure 4). However, variation in environmental pathogen load and in individual tolerance for the infection imply that the effect of an infection may vary substantially among individuals and environments. The proposed mixture models may be extended to allow for individual responses to infection (Madsen et al., 2008). Alternatively, the model may be extended to a growth mixture model (Wang and Bodner, 2007). The growth mixture models assume that the observations come from different latent trajectories, i.e., health status does not only affect the expectation of individual observations, but also the slope of a phenotypic trajectory (growth curves). Infected and non-infected animals could show different trajectories, with the non-infected ones being unaffected by the pathogen, while the infected individuals being variably affected by the pathogen burden. Such models may be useful to analyze resistance and infection-affected traits observed on animals with unknown infection status and in environments with variable pathogen loads.

### Application of the methods in breeding programmes

Random regression models are routinely applied in farm animal breeding programs, e.g., for milk test-day models in dairy cows and for growth curves (Schaeffer, 2004). Similarly, random regression models can be implemented to select for tolerance, given suitable data are available. The cure model approach for the analysis of time-until-death data (Veerkamp et al., 2001; Ødegård et al., 2011a) have been implemented in the DMU software, allowing the estimation of genetic parameters and breeding values for practical breeding (Madsen and Jensen, 2010). To our knowledge, the cure model has not been implemented in routine genetic evaluations in any breeding program. Árnason (1999) and Urioste et al. (2007) have proposed a bivariate linear-threshold model which can be used to analyze whether an animal survived (a threshold trait) and how long it took until death (a linear trait). Such a model resembles the cure model and is straightforward to apply in multi-trait breeding value evaluations. Also the normal mixture model has been implemented in the DMU software (Madsen and Jensen, 2010), and is therefore available for multi-trait genetic evaluations, but to our knowledge, has not yet been implemented in routine genetic evaluations.

The cure model has been applied to survival data in aquaculture species, leading to altered recommendations for routine disease-challenge testing (Ødegård et al., 2011b). Historically, challenge tests in aquaculture species have been terminated at intermediate cumulative mortalities to ensure maximum variation in binary survival data. However, this approach is only proper given that endurance and susceptibility are equivalent traits, which is not necessarily the case. The current advice is to continue testing until mortality naturally ceases, even at levels above 50% mortality (Ødegård et al., 2011a).

So far, only a limited number of breeding programs have considered selecting for tolerance. Some African cattle breeding programs are specifically selecting for trypanotolerance-related traits, the tolerance being a major breeding objective trait (Hanotte et al., 2003; van der Waaij et al., 2003). In contrast, regardless of the extensive studies conducted in Australia and New Zealand on nematode tolerance in sheep, a decision has been made not to record and select for tolerance because of the need to let animals to suffer and production to be reduced for tolerance to be expressed (Albers et al., 1987; Bisset and Morris, 1996; Woolaston and Windon, 2001). The novel statistical methods and the increasing awareness of the detailed physiological mechanisms of tolerance (Medzhitov et al., 2012) may provide more opportunities for tolerance selection in farm animals.

## Conclusions

The recent statistical developments provide tools to increase our understanding of genetics of alternative strategies to defend against parasites, pathogens, and production diseases. Most of the statistical methods can be applied in breeding value evaluations to breed for tolerance. Moreover, the methods presented here provide tools to quantify genotype-by-pathogen burden interactions that may explain a significant proportion of phenotypic variation in traits within populations that are exposed to various infections and production diseases. The traits whose variation is affected are typically production traits that are selected for in breeding programs. To be able to unambiguously select for the genetic potential of a production trait, the effects of resistance and tolerance should be separated from it. The methods presented in this paper provide potential to construct more effective breeding programs to increase both productivity and animal health.

### Conflict of interest statement

The authors declare that the research was conducted in the absence of any commercial or financial relationships that could be construed as a potential conflict of interest.
